# A randomized controlled trial of the efficacy of orally administered fluralaner (Bravecto™) against induced *Ixodes holocyclus* (Australian paralysis tick) infestations on dogs

**DOI:** 10.1186/s13071-015-0864-8

**Published:** 2015-05-01

**Authors:** Petr Fisara, Maurice Webster

**Affiliations:** MSD Animal Health, 26 Artisan road, Seven Hills, 2147 NSW Australia; Vetx Research, PO Box 23, Casino, NSW 2470 Australia

**Keywords:** Dog, Fluralaner, Tick, Paralysis, Australia, Ixodes holocyclus

## Abstract

**Background:**

*Ixodes holocyclus* ticks are a frequently fatal threat to dogs in eastern Australia. These ticks secrete a neurotoxin that can produce an ascending paralysis after 72 h attachment that can lead to death in affected animals. Fluralaner is a potent systemic acaricide with immediate and persistent efficacy for tick control including evidence of 100% efficacy against *Ixodes ricinus* ticks within 72 h. This study investigated the potential for oral fluralaner administration to control *I. holocyclus* infestation and the subsequent risk of host paralysis.

**Methods:**

Healthy Foxhound and Foxhound cross dogs immunized against holocyclotoxin were randomly allocated to receive either a single fluralaner (at least 25 mg/kg) dose or no treatment. All dogs were penned individually and infested with 30 adult unfed female *I. holocyclus* 1 day before treatment and 14, 28, 42, 56, 70, 84, 112 and 140 days following treatment. Ticks were counted and assessed at 24, 48 and 72 h after the initial fluralaner treatment and after each subsequent infestation. Ticks were not removed at the 24 and 48 h assessments, but were removed after the 72 h assessments. On 112 and 140 days post treatment a new group of untreated control dogs was used.

**Results:**

Fluralaner treatment efficacy against *I. holocyclus* was 100% at 72 h post treatment. Following re-infestations the efficacy remained at 100% at the 72 h assessments for 115 days and reached 95.7% at 143 days. The differences between mean live tick counts on treatment and control groups were significant (P < 0.00l) at all assessment time points for 143 days following treatment.

**Conclusions:**

Oral fluralaner treatment can prevent Australian paralysis tick infestations for at least 115 days.

## Background

The female of the *Ixodes holocyclus* tick secretes a potent holocyclotoxin following attachment that causes severe neurotoxicosis in man and domestic animals, including: dogs, cats, cattle, sheep, horses and goats. The neurotoxicosis is manifested as a rapidly ascending flaccid paralysis that can result in paralysis of respiratory muscles and death if not treated [1]. An estimated 10,000 domestic dogs are affected by *I. holocyclus* annually in Australia [2]; however, a recent study found that toy breeds were most at risk of tick paralysis associated death [3]. Most tick paralysis cases in dogs are reported in spring and early summer, although ticks and cases of paralysis may be found all year [3], and this seasonality corresponds with the presence of adult female ticks [4]. The *I. holocyclus* geographic range in eastern Australia extends from Lake Entrance in Victoria in the South, along the New South Wales and Queensland Coast to Cairns in the North. Tick distribution is based on several factors, and high humidity with low vegetation provide the ideal habitat for ticks [5]. Another factor affecting *I. holocyclus* prevalence is the bandicoot population as these animals are the most common natural hosts. Bandicoots are native marsupial species found on the eastern board of Australia. The most common species are the long-nosed bandicoot (*Perameles nasuta*) and the northern brown bandicoot (*Isoodon macroorus*); These small mammals often inhabit areas of coastal bushland and their presence is linked to the incidence of *I. holocycus* ticks [1].

Bandicoots can survive heavy tick infestations apparently because of an acquired immunity to the toxin rather than an intrinsic resistance [1].

Dogs at risk of tick attachment need protection against *I. holocyclus*, particularly when adult female ticks are present; however, pet owner compliance with tick control treatment application – even in the face of the risk of death from paralysis - remains a challenge. A prospective survey conducted at 42 veterinary clinics along the eastern coast of Australia showed that only 14% of all dogs presented at veterinary clinics with tick paralysis were correctly treated with a prophylactic tick control agent. Mortality in the surveyed dogs reached 5% despite the animals being treated in veterinary hospitals [6]. Therefore, currently registered preventative products are not achieving the goal of providing protection.

Understanding the relationship between feeding activity of *I. holocyclus* and the associated paralysis is important for understanding how this neurotoxicity can be prevented. *I. holocyclus* engorge in two phases with initial slow feeding over the first 72 h after attachment and then a more rapid phase after 120 h. The swelling from the initial slow feeding can be obvious from as early as 10 minutes after attachment, when the tick changes from a flattened dorso-ventral appearance to having a slightly turgid appearance. At 24 h post attachment the ticks are not noticeably wider, but appear slightly swollen. Ticks continue swelling over the following 48 h, and by 72 h post attachment are approximately 30% of the volume of a fully engorged tick and show a rectangular block shape. The end of the engorgement process is marked with a dramatic engorgement just prior to detachment after approximately 7 days [7].

Toxin secretion has a specific timing during the engorgement process. Studies on factors affecting salivary gland extract toxicity in a mouse bioassay found that toxin quantity increased rapidly from the third day after feeding, apparently associated with major physiological changes in tick salivary glands occurring on the third day. Once these changes are stimulated, they continue independently of further tick feeding [8]. Onset of paralysis is never seen before the end of the fourth, or beginning of the fifth day after attachment [9]. Laboratory dogs infested with 3 to 4 ticks had an onset of clinical signs of toxicity between 5.5 and 7 days (mean = 6.2) after attachment with death occurring in a mean of 23.3 h after the onset of signs [10]. Therefore, to prevent the occurrence of fatal paralysis in infested dogs, it is critical to kill the ticks within 72 h post infestation and prior to the onset of clinical signs. Paralysis in dogs can be avoided if ticks are found and removed 1-2 days after attachment, however, once clinical signs begin to manifest from the fourth day onwards the paralysis may be fatal if left untreated even if the tick(s) are removed.

Fluralaner (Bravecto, MSD Animal Health) has proven to have immediate and persistent efficacy against multiple genera and species of ticks that infest dogs in Europe and in the USA [11,12]. Fluralaner is orally administered and is systemically effective against ticks and therefore the tick must attach and initiate feeding to be exposed to the active ingredient. However, the onset of activity of fluralaner against ticks results in greater than 90% kill of I. *ricinus* ticks within 12 h throughout the 12 week post-treatment interval [13] and tick mortality was found to reach 100% within 12 h following the initial treatment [14]. This speed of kill suggests that fluralaner treatment has the potential to effectively prevent tick paralysis by killing *I. holocyclus* ticks before they begin to secrete increased amounts of holocyclotoxin which occurs after the third day of feeding [8]. This study was designed to measure the efficacy of fluralaner treatment against *I. holocyclus* using dogs hyper-immunized against holocyclotoxin. The study was conducted in compliance with the VICH GL9 Good Clinical Practices [15], the World Association for the Advancement of Veterinary Parasitology (W.A.A.V.P.) Guidelines for Evaluating the Efficacy of Parasiticides for the Treatment, Prevention and Control of Flea and Tick Infestation on Dogs and Cats [16] and the APVMA Guidelines for small animal ectoparasiticide efficacy submission [17]. Animals were handled in compliance with Animal Research Authority no. 12/544(23) issued by the AEC convened by the Director General of NSW DPI on 6 February 2012, and any applicable local regulations.

## Methods

Twenty four healthy male and female dogs between 1.0 and 10.8 years old that had not been treated with an ectoparasiticide in the previous 60 days were immunized against holocyclotoxin, the tick paralysis toxin. All dogs were infested with *I. holocyclus* prior to random allocation to ensure that they were capable of carrying adequate numbers of ticks. The 20 dogs found to have the highest tick carrying capacity were randomly assigned to either the treatment or control groups using a randomized block design based on pre-treatment live tick counts. Each dog was weighed once prior to treatment and then dogs in the treated group received orally administered fluralaner (Bravecto, MSD Animal Health) at a minimum dose of 25 mg/kg in a flavoured chewable tablet. The dose for each dog was based on the chewable tablet size recommended for the weight band that the dog fell into. The individual doses ranged between 25.8 and 35.0 mg/kg fluralaner. This is lower than the maximum possible dose (56 mg/kg) administered to a dog treated according to the package insert. Following fluralaner treatment administration the animals were offered food and were closely observed for adverse reactions.

The study protocol was designed to end at 87 days following the initial treatment; however, efficacy remained high and the study was extended for a further 8 weeks. Some dogs in the original control group could not continue in the extended study because of previous unrelated commitments. Therefore, a new control group was created by combining 4 of the original 10 untreated control dogs with 4 new untreated immunized dogs. There were no changes made to the treated group. All dogs in the extended study period were tick challenged 2 more times on days 112 and 140.

Adult unfed female *I. holocyclus* were collected from the Northern Rivers region of New South Wales. All dogs in the experiment, including the second control group, were immunized against holocyclotoxin using a modification of the methods described by Stone, Neish & Wright [18]. At the end of this process, dogs were able to tolerate a challenge of 30 ticks with no evidence of intoxication. For each tick infestation during the experiment, 30 ticks were manually attached to each dog predominantly on the head, shoulders and dorsal midline. Infestations were applied 1 day before the treatment date and 14, 28, 42, 56, 70, 84, 112 and 140 days following treatment. During the tick challenge dogs were housed individually, while at all other times they were housed in socially compatible groups of 2 or 3 dogs from the same experimental groups. All dogs survived the study and were returned to their original colony on study completion.

The same personnel conducted all tick counts to ensure a standardized technique during assessments. Tick counting personnel wore disposable overalls to avoid skin contact with treated dogs and thoroughly washed their hands with non-acaricidal soap after assessing each dog and between counts on groups of dogs to ensure no possible transfer of active ingredient. Tick counts were conducted 24, 48 and 72 h after each infestation by searching the whole body of the dog visually and by palpation until no more ticks could be found. The 24 and 48 h assessments were conducted without removing ticks, while at the 72 h assessments any remaining ticks were removed, assessed and discarded. All dogs were searched again prior to each infestation to ensure that no ticks had been missed during the previous tick assessment. Each tick observed during the count was described according to selected parameters (Table 1). Some ticks were classified as ‘dead’ when examined *in situ* at 24 and 48 h but displayed uncoordinated agonal leg movement after removal at 72 h and were then reclassified as “moribund”. These ticks typically exhibited reduced engorgement and were smaller than live ticks of the same age with evidence of mild crenation (slightly shriveled appearance). These ticks were included in the total dead tick count as they were not feeding and were not capable of causing paralysis in the dogs.

**Table 1:** **Parameters used to describe observed ticks at each assessment**

**Parameter**	**Choices**	**Comments**
Viability	Live, Dead	Live ticks showed leg movement, no crenation and possible engorgement, presence of tick faeces and/or attachment site inflammation. Dead ticks had no leg movement, no reaction when stimulated, and possible crenation.
Attachment	Attached, Free	A tick classified as attached had its hypostome embedded into the dog’s skin and was not easily dislodged. A free tick was either live and moving through the coat, or dead and sitting in the hair.
Feeding	Unengorged, Partially Engorged or Fully Engorged	\Unengorged ticks showed no swelling or evidence of blood ingestion; engorged ticks were 12 - 15 mm long, 8- 10 mm wide and had a turgid appearance. Engorged ticks are typically not seen before 6 days post infestation and were not observed during this study because ticks were removed at 72 h post infestation. Engorging ticks show a conspicuous swelling of the alloscutum, and appear wider and longer than unengorged ticks. These ticks also contain blood or digested blood.

The recorded description of each tick was then used to place it in one of seven categories (Table 2). At each counting period the total number of ticks counted on each dog that were assigned to categories 1, 2, 3 & 7 were used in the calculation of the results.

**Table 2:** **Tick category assignments used to assess the acaricidal effect of treatment**

**Category**	**Observations**	**Acaricidal effect**
1	Live, Free, Unattached	No
2	Live, Attached, Not Engorged	No
3	Live, Attached and Engorging or Engorged	No
4	Dead, Free	Yes
5	Dead, Attached, Not Engorged	Yes
6	Dead, Attached, Engorging	Yes
7	Dead, Attached, Engorged	No

Treatment efficacy was calculated using arithmetic means as:

$$ \mathrm{Treatment}\ \mathrm{efficacy} = 100*\left(\mathrm{Control}\ \mathrm{Group}\ \mathrm{Mean}\hbox{--}\ \mathrm{TreatedGroup}\ \mathrm{Mean}\right)/\left(\mathrm{Control}\ \mathrm{Group}\mathrm{Mean}\right) $$

The experimental unit was the individual animal; very few ticks were counted on treated dogs for most of the study and block differences were negligible, therefore, they were not included in the analyses. Generalized linear models for Poisson data using the logarithmic link function (log-linear modelling or regression, or Poisson regression) [19] was used to compare mean total live tick counts for dogs in control and treated groups at each post-treatment sampling occasion. This method produces an analysis of deviance, analogous to the analysis of variance for normally distributed data. In the generalized linear models method, the observed counts are analyzed and the mean-variance relation is used to link mean counts to the linear model on the logarithmic scale [20].

## Results and discussion

Mean tick counts at each sampling point (Table 3) show that the tick infestation level on control dogs was sufficient and that trial results can be used to confirm treatment efficacy. Mean tick counts on treated dogs were significantly lower than the mean tick counts on control group dogs at each time point post-infestation and post-treatment (Table 3). Mean tick counts on treated dogs at 24 h post-infestation were below 1.0 until 113 days post-treatment (Table 3). The 72 h post-infestation mean tick counts were 0 (and therefore an efficacy of 100% was achieved ) at any measurement point prior to the final count at 143 days post treatment.

**Table 3:** **Arithmetic mean** ***I. holocyclus*** **tick counts in fluralaner treated and untreated dogs**

**Time following treatment (d)**	**Time after treatment or re-infestation (h)**	**Control**	**Treated**	**P**
1	24	23.6	0.1	<0.001
2	48	23.7	0.0	<0.001
3	72	23.0	0.0	<0.001
15	24	25.7	0.1	<0.001
16	48	24.8	0.0	<0.001
17	72	24.8	0.0	<0.001
29	24	25.8	0.1	<0.001
30	48	25.7	0.2	<0.001
31	72	25.7	0.0	<0.001
43	24	26.7	0.8	<0.001
44	48	26.8	0.0	<0.001
45	72	27.0	0.0	<0.001
57	24	26.1	0.1	<0.001
58	48	25.8	0.0	<0.001
59	72	25.6	0.0	<0.001
71	24	25.4	0.2	<0.001
72	48	25.3	0.0	<0.001
73	72	25.1	0.0	<0.001
85	24	26.4	0.3	<0.001
86	48	25.6	0.2	<0.001
87	72	25.6	0.0	<0.001
113	24	27.1	3.5	<0.001
114	48	26.6	0.1	<0.001
115	72	26.5	0.0	<0.001
141	24	26.4	14.4	<0.05
142	48	25.9	5.7	<0.01
143	72	25.5	1.1	<0.001

**Figure 1:**
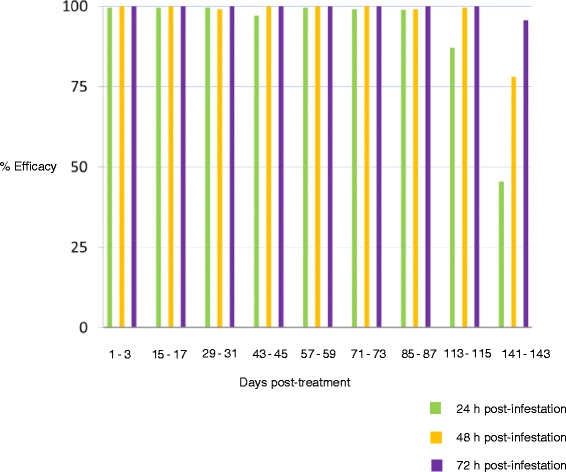
Acaricidal efficacy of fluralaner treatment of dogs against adult *Ixodes holocyclus* ticks.

Efficacy results (Figure 1) show that fluralaner treatment provided 100% efficacy at 72 h following induced *I. holocyclus* infestation of dogs for 115 days post treatment. Onset of paralysis following *I. holocyclus* attachment is never seen before the end of the fourth, or beginning of the fifth day after attachment [9]; therefore, the 100% efficacy at 72 h found in this study means that all attached adult female ticks are killed before the onset of clinical signs. It should be noted that due to the systemic nature of action of fluralaner, without any apparent repellency effect, dead attached ticks can be still seen on animals following treatment. These ticks will often appear partially engorged and can be easily mistaken for live ticks by an untrained person. This could be perceived as a lack of efficacy by pet owners, however, fluralaner relies on the parasite to ingest the medicated blood during feeding to become affected and killed. Dead ticks can be easily removed as opposed to live ticks which take some force to dislodge from the skin of the animals. Despite the fact that attached ticks were seen on the dogs at post treatment counts in this trial, all of these ticks were killed well within the critical period of 72 hours after attachment before paralysis begins to set in. Consequently, pet owners will need to be educated about the way fluralaner works and protects their dogs from tick paralysis so that they are not unduly alarmed by a dead attached tick.

It is assumed that fluralaner oral treatment can, under the conditions of this study, effectively prevent the occurrence of fatal paralysis for 115 days. Additionally, fluralaner treatment continued to significantly reduce the number of ticks at 72 h post infestation for 143 days, reaching 95.7% at the last measurement time point in this study. This is a uniquely persistent efficacy against this dangerous parasite following a single administration of a systemic treatment.

Lack of pet owner compliance with recommendations for topical treatment [6] has limited the ability of veterinarians to prevent tick paralysis in Australian dogs. Therefore, the formulation of fluralaner into a palatable and easily administered tick control treatment provides an important new option for veterinarians and owners in preventing the neurotoxicosis associated with *I. holocyclus*. Palatability, defined as voluntary acceptance of the chewable tablet following administration to the dog by the owner at home, was reported to be 92.5% [12].

No treated dog vomited the administered medication during the post treatment period in this study, which is consistent with results observed in an extensive field trial in Europe [11]. However, there were 3 adverse events reported in untreated dogs and 2 in treated dogs during this study. Adverse events in treated dogs included one dog that developed otitis externa 79 days after treatment and one dog that developed forelimb lameness 95 days after treatment. The timing and nature of these 2 events was such that neither adverse event was considered to be related to fluralaner administration.

## Conclusions

Oral fluralaner treatment of dogs kills 100% of attached *I. holocyclus* adults within 72 h post infestation for at least 115 days and can be used to prevent the onset of clinical signs of tick paralysis. The tick killing effect of 95.7% is still present at 143 days post administration.
